# The incidence and predictors of in-stent stenosis after pipeline flow-diverter stenting for intracranial aneurysm treatment

**DOI:** 10.3389/fneur.2023.1140497

**Published:** 2023-04-25

**Authors:** Wei You, Jian Lv, Zifan Li, Xiheng Chen, Dingwei Deng, Yudi Tang, Youxiang Li, Yong Sun, Yuhua Jiang

**Affiliations:** ^1^Department of Neurosurgery, Beijing Neurosurgical Institute, Beijing Tiantan Hospital, Capital Medical University, Beijing, China; ^2^Syracuse University, Syracuse, NY, United States; ^3^Beijing Engineering Research Center, Beijing, China; ^4^Department of Neurosurgery, The First People's Hospital of Lianyungang, Affiliated Hospital of Kangda College of Nanjing Medical University, Lianyungang, China

**Keywords:** flow diverter, intracranial aneurysm, stent, pipeline, in-stent stenosis (ISS)

## Abstract

**Background and purpose:**

Data on in-stent stenosis (ISS) following the flow diverter (FD) implantation method are scarce and inconsistent. In the present study, we sought to determine the incidence of ISS and identify the factors that predict its severity via the use of ordinal logistic regression.

**Methods:**

A retrospective review of our center's electronic database was conducted to identify all patients with intracranial aneurysms (IAs) who received pipeline embolization device (PED) implantation between 2016 and 2020. Patient demographics, aneurysm characteristics, procedural information, and clinical and angiographic outcomes were reviewed. ISS was quantitatively assessed on angiographic follow-ups and graded as mild (<25%), moderate (25–50%), or severe (>50%). Ordinal logistic regression was conducted to determine the predictors of stenosis severity.

**Results:**

A total of 240 patients with 252 aneurysms treated in 252 procedures were enrolled in this study. ISS has been detected in 135 (53.6%) lesions, with a mean follow-up time of 6.53 ± 3.26 months. The ISS was mild in 66 (48.9%) cases, moderate in 52 (38.5%) cases, and severe in 17 (12.6%) cases. All patients were asymptomatic, except for two of them with severe stenosis who presented with symptoms of acute cerebral thrombosis. Ordinal logistic regression identified that younger age and a longer procedure duration were independent predictors of a higher likelihood of ISS.

**Conclusion:**

ISS is a common angiographic finding after PED implantation for IAs and is presented as a largely benign course through long-term follow-up. Patients who were younger in age and had a longer procedure duration were found to be at a greater risk of developing ISS.

## Introduction

Having gained widespread global acceptance, flow diverters (FDs) have revolutionized the treatment of IAs (1). The pipeline embolization device (PED) is one of the earliest and most widely used FDs, and its efficacy and safety have been confirmed (2). Many previous studies have reported occlusion rates and hemorrhagic or ischemic complications after FD implantation (3, 4). However, data on the incidence and predictors of in-stent stenosis (ISS) after FD implantation are scarce and confusing (5–8).

In-stent stenosis is generally defined as a loss of vessel diameter found on follow-up DSA imaging and is associated with pathophysiological changes after stent implantation (9, 10). The definition of ISS after FD implantation is not well established, as some scholars use different diagnostic criteria, such as >50 or 25% stenosis. To the best of our knowledge, to date, at least three different judgment criteria have been reported in the literature (6, 11, 12). Furthermore, the wide range of ISS occurrences reported in the literature, from 0% (12) to 100% (13), is a result of these inconsistent standards. This lack of a clear definition makes it difficult for us to understand and summarize research findings and may result in the definition of ISS changing in the future.

Although most cases of ISS are asymptomatic, some progress and cause serious complications (14, 15). A reliable method for identifying predictors that are significantly associated with ISS severity is essential. However, to date, most studies have relied on dichotomous rather than ordered categorical data in their statistical analyses (16, 17). It is well known that ignoring orders has its own disadvantages, mainly because it does not fully utilize the available information (18).

In this study, we evaluated the incidence of ISS in patients with IAs who were treated with PED at our center. ISS was defined as any discernible gaps between contrast-filled vessels and metallic struts present in angiographic follow-up images. Ordinal logistic regression was used in the present study to determine the factors associated with the severity of ISS. Our research was a single-center study with a large cohort of patients who underwent PED treatment for IAs. Our findings may provide valuable insights for both doctors and patients into this phenomenon.

## Methods

### Study population

We conducted a retrospective review of patients with intracranial aneurysms who received PED treatment in the Interventional Neuroradiology department of our hospital between 2016 and 2020. Patients with at least one digital subtraction angiography (DSA) follow-up and without PED implantation failure were enrolled in this study. Patient demographics, aneurysm characteristics, procedural information, and clinical and angiographic outcomes were reviewed. This retrospective study was approved, and patients' written consent was waived by our institutional review board.

### Endovascular procedure

The patients received dual antiplatelet medication consisting of aspirin 100 mg/day and clopidogrel 75 mg/day for 7 days before the implantation. Routine preoperative platelet function tests were performed, and patients who were identified as clopidogrel non-responders were given either prasugrel or ticagrelor. All PED implantations were performed under general anesthesia *via* a femoral approach. According to the aneurysm anatomy and the operator's experience, the treatment strategy was formulated based on the decision of whether PED alone or PED plus coiling would be used. After the procedure, the patients were prescribed dual antiplatelet therapy for 6 months, with aspirin being continued indefinitely thereafter. Clinical follow-ups were conducted at 3, 6, 12, and 24 months after the treatment.

### Angiographic evaluation of ISS

**Figure 1:**
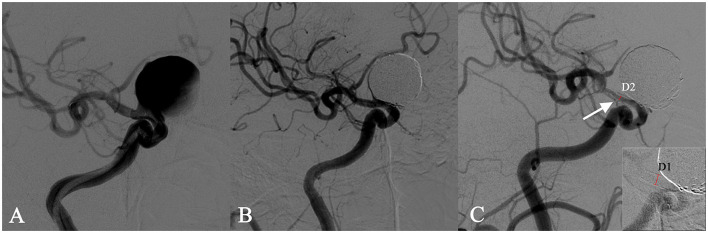
In-stent stenosis in a man in his 40s who presented with a symptomatic right carotid ophthalmic aneurysm **(A)** and was treated with the implant of a single PED stent plus coiling. Angiographic images obtained immediately after the intervention showed an unimpeded flow in the stent **(B)**. The follow-up angiography after 6 months showed a 70% in-stent stenosis (ISS% = 1 – [D2/D1] × 100%) at the distal end of the stent **(C)**.

ISS is defined as any reduction in the parent artery filled with contrast medium at a follow-up DSA. In DSA, ISS is shown as a discernible gap between the vessel lumen filled with contrast medium and the inner wall of PED. Moreover, cases with no discernible gap in follow-up DSA were excluded from this study. For discernible gaps, we measured the diameter of the contrast-filled vessel and the endovascular stent diameter at its corresponding position. The rate of stenosis was then calculated as the ratio of the contrast-filled vessel diameter to the endovascular stent diameter, expressed as a percentage (Figure 1). For the diffuse ISS, we selected the maximum stenosis percentage as the representative value for analysis in the study. ISS was then graded as mild (<25%), moderate (25–50%), and severe (≥50%). In addition, ISS was divided into focal and diffuse lesions based on the location of the stents (proximal, middle, and distal), whether they extended more than 10 mm, and whether they were located at a vessel curvature. The assessment and measurement of ISS through DSA follow-up images were performed by neuroradiologists with at least 3 years of experience and reviewed by a senior neuroradiologist.

### Statistical analysis

The data were presented as frequencies for categorical variables and as means and ranges for continuous variables. Unpaired *t*-tests, Chi-squared tests, and Fisher's exact tests were conducted to assess variable differences. Ordinal logistic regression was used to determine the factors associated with the severity of ISS. Variables that were found to be significant at a level of 0.1 under crude association analysis or based on clinical relevance were entered into the multiple logistic regression analysis. The results were presented as odds ratios (OR) and corresponding 95% confidence intervals (CI). A *p*-value of < 0.05 was considered statistically significant. All statistical analyses were carried out using the SPSS version 22.0.0 software (IBM, Armonk, NY, USA).

## Results

### Patient demographics, aneurysm characteristics, procedure details, and clinical outcomes

A total of 240 patients (mean age: 50.9 ± 12.8 years; 157 women, 65.4%) with 252 aneurysms treated through PED implantation in 252 procedures and with at least one DSA follow-up were included in this study. The demographic, baseline, and procedural characteristics of the cohort are presented in Table 1. Comorbidities included hypertension, diabetes, hyperlipidemia, coronary artery disease, a history of allergies, alcohol abuse, and smoking. The aneurysms were found incidental in 68 cases (28%), symptomatic in 184 cases (73%), and ruptured in 16 cases (6.3%). A total of 8 aneurysms (3.2%) were treated previously, that is, two that were treated with coiling and six that were treated with stent-assisted coiling.

**Table 1:** Univariate and ordinal logistic regression analyses in relation to the severity of stenosis.

**Variables**	**Frequency (%)**	**Non or Mild ISS**	**Moderate ISS**	**Severe ISS**	**Univariate** ***p***	**Multivariate**	
						***p***	**OR (95% CI)**
**Baseline demographics and clinical characteristics**							
Women, no. (%)	168 (66.7%)	130 (71%)	32 (61.5%)	6 (35.3%)	0.01^*^	0.4	0.70 (0.31-1.6)
Age, y (mean ± SD)	50.98 ± 12.69	52.31 ± 11.8	48.77 ± 14	43.41 ± 14.83	0.004^*^	0.02†	0.97 (0.95-1)
BMI	25.03 ± 4.17	24.96 ± 3.63	25.14 ± 5.72	25.42 ± 4.38	0.68		
**Comorbidities**							
Hypertension, no. (%)	101 (40.1%)	77 (42.1%)	17 (32.7%)	7 (41.2%)	0.34		
Diabetes, no. (%)	19 (7.5%)	12 (6.6%)	4 (7.7%)	3 (17.6%)	0.24		
Hyperlipidemia, no. (%)	89 (35.3%)	65 (35.3%)	17 (32.7%)	7 (41.2%)	0.98		
Coronary artery disease, no. (%)	22 (8.7%)	19 (10.4%)	2 (3.8%)	1 (5.9%)	0.15		
History of allergies, no. (%)	36 (14.3%)	30 (16.4%)	6 (11.5%)	0 (0%)	0.1		
Smoking, no. (%)	49 (19.4%)	30 (16.4%)	12 (23.1%)	7 (41.2%)	0.03^*^	0.18	1.82 (0.76-4.38)
Alcohol abuse, no. (%)	52 (20.6%)	34 (18.6%)	14 (26.9%)	4 (23.5%)	0.21		
Symptomatic presentation of IA, no. (%)	184 (73%)	142 (77.6%)	31 (59.6%)	11 (64.7%)	0.25		
Ruptured (history of SAH), no. (%)	16 (6.3%)	10 (5.5%)	5 (9.6%)	1 (5.9%)	0.41		
Previous treatment of IA, no. (%)	17 (6.75%)	13 (7.1%)	3 (5.8%)	1 (5.9%)	0.72		
**Aneurysm characteristics**							
Saccular aneurysm, no. (%)	201 (79.8%)	153 (60.7%)	37 (14.7%)	11 (4.4%)	0.01^*^	0.1	0.47 (0.19-1.17)
Aneurysm neck size (mm)	9.29 ± 5.84	9.04 ± 5.85	9.23 ± 5.06	12.22 ± 7.45	0.15		
Maximum diameter (mm)	13.45 ± 7.89	13.34 ± 8.16	13.01 ± 6.42	15.65 ± 9.14	0.15		
Parent artery diameter (mm)	3.69 ± 0.95	3.76 ± 0.93	3.92 ± 0.87	2.86 ± 1.01	0.02^*^	0.09	0.76 (0.55-1.05)
Associate with parent artery stenosis, no. (%)	20 (8%)	11 (6%)	4 (7.7%)	5 (29.4%)	0.02^*^	0.19	1.9 (0.72-4.98)
Bifurcation aneurysm, no. (%)	16 (6.3%)	8 (4.4%)	5 (9.6%)	3 (17.6%)	0.03^*^	0.18	2.05 (0.72-5.81)
Anterior circulating aneurysm, no. (%)	193 (76.8%)	146 (71.2%)	37 (58.8%)	10 (76.6%)	0.04^*^	0.3	1.64 (0.65-4.15)
**Procedure characteristics**							
PED plus coiling, no. (%)	119 (47.2%)	86 (47%)	24 (46.2%)	9 (52.9%)	0.85		
Pipeline Flex embolization device, no. (%)	140 (55.6%)	107 (58.5%)	25 (48.1%)	8 (47.1%)	0.13		
Multiple PED implantations, no. (%)	43 (17.1%)	31 (16.9%)	8 (15.4%)	4 (23.5%)	0.85		
Balloon angioplasty, no. (%)	52 (20.6%)	40 (21.9%)	6 (11.5%)	6 (35.3%)	0.61		
Procedure duration (min)	120.93 ± 53.68	117.34 ± 51.15	123.44 ± 60.9	151.88 ± 49.4	0.05^*^	0.01^†^	1.01 (1-1.01)

BMI, body mass index; IA, intracranial aneurysm; SAH, subarachnoid hemorrhage; PED, pipeline embolization device.

The

* symbol represents statistical significance (p < 0.05) in univariate analysis,

while the

† symbol represents statistical significance (p < 0.05) in multivariate analysis.

In total, 201 (79.8%) saccular and 51 (20.2%) fusiform aneurysms were identified. Most of the aneurysms were located in the internal carotid artery (184/252, 73%), with 47 (18.7%) found in the vertebral arteries, 12 (4.8%) in the basilar and other posterior cerebral arteries, and 9 (3.6%) in the distal circle of Willis (including the middle cerebral artery, anterior cerebral artery, and communicating artery). Of the 252 aneurysms, 16 (6.3%) were located at a bifurcation, and 193 (76.8%) were located in the anterior circulation. The mean aneurysm neck size of the aneurysms was 9.29 ± 5.84 mm, the mean maximum diameter was 13.45 ± 7.89 mm, and the mean parent artery diameter was 3.69 ± 0.95 mm. Moreover, 20 (8%) aneurysms were associated with parent artery stenosis.

In total, 140 (55.6%) procedures were performed with the Pipeline™ Flex embolization device, while the remaining were performed using the Pipeline™ Classic embolization device. Of the 252 procedures, 133 (52.8%) were treated using PED alone, and 119 (47.2%) were treated using a combination of PED and coiling. PED was deployed successfully in all patients. Multiple PED implantations were performed in 43 (17.1%) procedures, and balloon angioplasty was administered in 52 (20.6%) procedures. The mean procedure duration was 120.93 ± 53.68 min.

At the last angiographic follow-up examination, complete aneurysm occlusion was observed in 213 cases (84.5%). The rates of periprocedural ischemic complications (periprocedural stroke or transient ischemic attacks) and hemorrhage complications were 2.8% (7/252) and 0.8% (2/252). Transient deficits were observed in 8 (3.2%) cases, and permanent deficits (mRS > 2) were observed in 4 (1.6%) cases. There were no cases of periprocedural mortality.

### In-stent stenosis

In-stent stenosis was detected in 135 (53.6%) lesions using the quantitative assessment. All stenoses were detected at the first DSA follow-up, with a mean time of 6.53 ± 3.26 months. ISS was mild in 66 (48.9%) cases, moderate in 52 (38.5%) cases, and severe in 17 (12.6%) cases. The stenosis was diffuse in 56 (41.5%) cases and focal in 79 (58.5%) cases. There were 47 (34.8%) occurrences of stenosis located at the proximal end of the stent, 52 (38.5%) in the middle, 36 (26.7%) at the distal end, and 48 (35.6%) at the bend of the artery.

**Figure 2:**
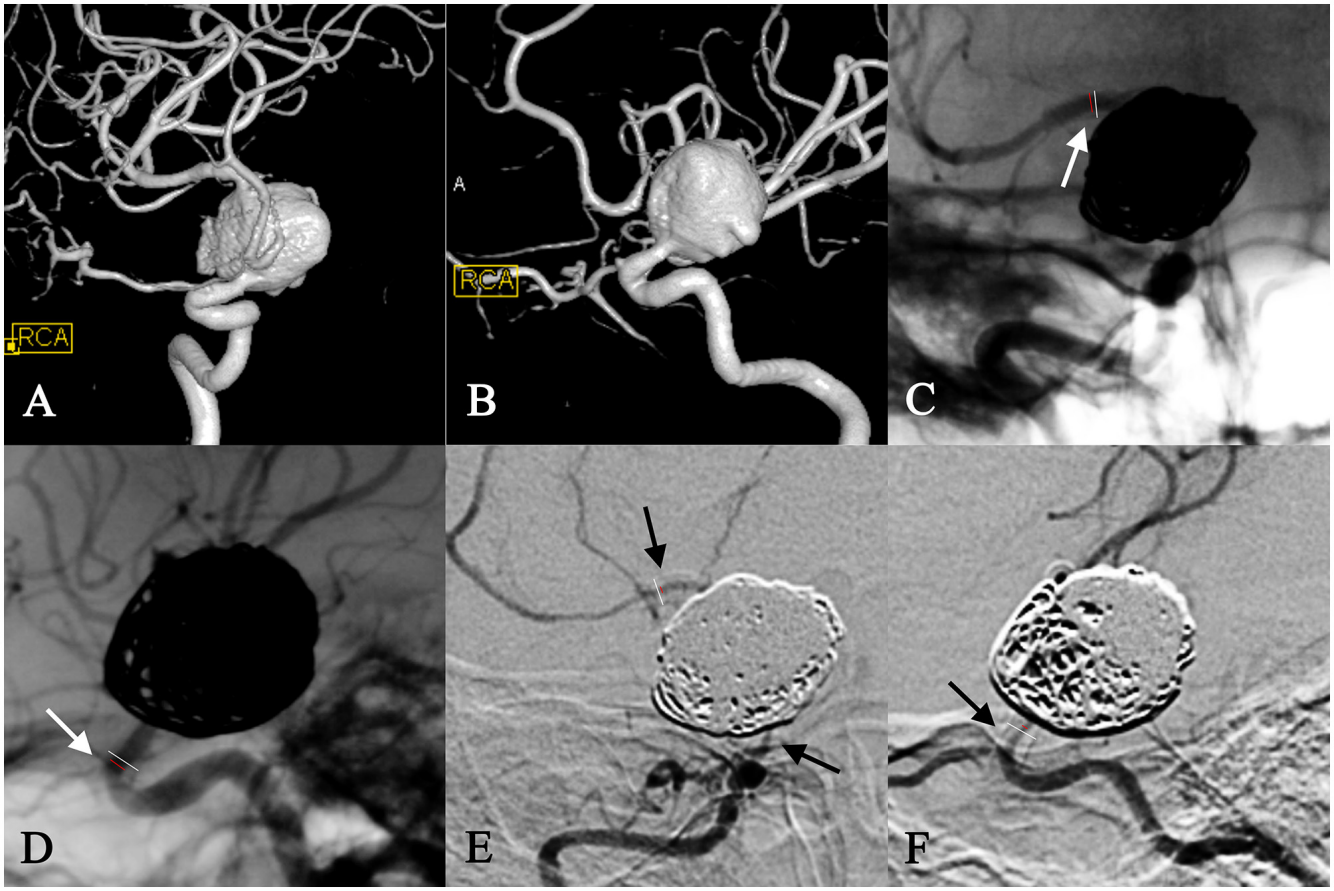
In-stent stenosis in a woman in her 30s with a right carotid ophthalmic aneurysm **(A, B)** and treatment with PED plus coiling. At the 6-month follow-up, the frontal view of the angiography showed 15% in-stent stenosis at the distal end of the stent **(C)**, and the lateral view of the angiography showed 65% stenosis at the proximal opening of the stent **(D)**. This patient developed left hemiplegia due to a right cerebral infraction 10 months after surgery, which was relieved by a thrombolysis at the local hospital. ISS in this case aggravated to 90% diffuse stenosis at the 18-month follow-up **(E, F)** and was subsequently treated by a vascular bypass between the superficial temporal artery and middle cerebral artery.

While most cases were asymptomatic, symptomatic stenosis was identified in two cases. One patient who was treated for a right carotid artery aneurysm with 65% stenosis at the 3-month follow-up showed left hemiplegia, which was caused by a right cerebral infarction 10 months after treatment; symptoms of the infarction were relieved by thrombolysis at the local hospital. The stenosis, in this case, had aggravated to 90% by the 18-month follow-up and was subsequently treated by vascular bypass between the superficial temporal artery and the middle cerebral artery (Figure 2). The other patient had a left middle cerebral aneurysm and suddenly showed combined aphasia, which was caused by 95% stenosis accompanied by stent thrombosis at the 6-month follow-up. The patient's symptoms resolved, and 80% stenosis remained after further treatment with balloon angioplasty and stent thrombectomy.

Among the 135 patients with ISS, 21 (15.6%) of them had long-term angiographic follow-ups with a mean time of 25.1 ± 9.4 months. Of the 21 cases, 8 (38.1%) showed completely resolved stenosis, 4 (19%) were in remission, 7 (33.3%) were stable, and 2 (9.5%) showed progress. In addition to the aforementioned cases of stenosis progression, the other case had aggravated from mild stenosis (19%) to moderate stenosis (37%) with no symptoms.

All cases were classified into three ordinal forms according to the likelihood of stenosis: non- or mild ISS, moderate ISS, and severe ISS. In the crude association analysis, significant predictors of ISS severity included female gender (*p* = 0.008), age (*p* = 0.004), smoking status (*p* = 0.03), saccular aneurysm (*p* = 0.01), parent artery diameter (*p* = 0.02), associated with parent artery stenosis (*p* = 0.02), bifurcation aneurysm (*p* = 0.03), anterior circulating aneurysm (*p* = 0.04), and procedure duration (*p* = 0.05). These factors were found to be significant at the level of 10% and were entered as subsets in ordinal logistic regression. In the multivariate regression analysis, the overall proportionality assumption was not violated (*p* = 0.13). Ordered logistic regression analysis showed that age and procedure duration were significant predictors of a higher likelihood of stenosis after PED implantation. To be specific, the cases with a longer procedure duration (OR = 1.01; 95% CI, 1–1.01; *p* = 0.012) had a higher likelihood of developing stenosis, whereas cases with older patients (OR = 0.97; 95% CI, 0.95–1; *p* = 0.017) had a lower likelihood of stenosis (Table 1).

## Discussion

In the present study, we reported that 53.6% of the lesions had radiographically identifiable ISS, 27.38% had more than 25% stenosis, and 6.75% had more than 50% stenosis. Ordinal logistic regression was used to determine the significant factors associated with the severity of ISS. The multivariate analysis revealed that a longer duration of the procedure and a younger age were independent predictors of a higher likelihood of stenosis.

Previous literature has reported highly differentiated ISS rates ranging from 0.61 to 43.75% after PED implantation (7, 8, 19–21). This wide range is likely due to the different definitions and grading standards of the ISS that have been used by different authors. Unlike the clear definition of in-stent restenosis after coronary stent implantation, there is variable phrasing for the same postoperative imaging findings, such as “in-stent stenosis” (13) or “neointimal hyperplasia.” (20). Although some researchers believe that ISS should be derived from neointimal hyperplasia (7, 14), there is currently no consensus on the specific criteria for determining the likelihood of ISS. Caroff et al. (20) considered all degrees of the vascular lumen reduction to be neointimal hyperplasia. John et al. (6) considered neointimal hyperplasia as the narrowing of the vessel of <25% and ISS as narrowing of more than 25%. Additionally, some authors considered ISS as vessel stenosis of more than 50% (22), and some did not clarify the criteria (8, 23). The vagueness and differentiation of definitions of ISS after IA stent treatment in previous literature have made comparisons difficult.

ISS is a well-known issue in endovascular stent implantation, especially in the treatment of coronary arteries, and has been described with conventional intracranial aneurysm stents in previous studies (17, 24). The underlying cellular mechanisms of ISS have not been well described but may be associated with platelet activation and inflammation in the early phase, endothelialization and granulation tissue formation in the intermediate phase (9, 25) and smooth muscle cell and matrix formation in the late phase. Intra-aneurysmal thrombosis and the migration of endothelial cells across the aneurysmal neck along the scaffold are two major processes during aneurysm occlusion using FD (26). Therefore, considering the mechanism of aneurysm occlusion, mild stenosis, which has been defined in other studies as neointimal growth, is to be expected. This is the reason the cases with no stenosis and the cases with mild stenosis were classified at the same level in the ordinal logistic regression.

In biomedical research, sometimes, ordered categories are the result of quantitative data grouping, in addition to the frequent occurrence of ordinal categorical data. Although previous studies have classified ISS in different grades, stenosis has only been discussed in the dichotomous form, not the ordinal form (16, 17). Their results are limited by not taking full advantage of the available information. To date, no study has considered the ordinal form of ISS severity when assessing its associated factors. In the present study, ordinal logistic regression was used to determine the factors associated with the severity of ISS.

A possible explanation for younger patients being more likely to have a higher likelihood of stenosis is that the neointimal response induced by stent implantation is more robust in younger patients. Du et al. (27) confirmed this finding by observing a significant reduction of in-stent neointimal growth after coronary stenting in older patients compared with younger patients. Additionally, our finding is also consistent with the study by Chalouhi et al. (17), who found that younger age is an independent factor for ISS after stenting with Neuroform and Enterprise.

We included the procedure duration as a new variable in this study, which had not been considered in previous studies. Surprisingly, we found that a longer procedure duration was an independent predictor of a higher likelihood of stenosis. It is clear that a longer procedure duration results in a relatively higher number of operations being required, which in turn causes more damage to the endothelium. The deployment and adjustment of the stent and balloon usage inevitably result in endothelial injury. In the absence of functional endothelial cell regulation, regional smooth muscle cells activate and proliferate, resulting in neointimal tissue formation, which leads to ISS (28).

It is worth noting that the parent artery diameter may also affect the occurrence and development of stenosis. Although artery diameter was not a significant predictor of stenosis severity, it has been identified as a predictor of restenosis after coronary stenting (11). Compared with larger-diameter arteries, such as the carotid artery, the luminal diameter of the smaller vessels was dramatically influenced by intimal hyperplasia (29). Smoking has been identified as an important factor of ISS in previous studies (8) and showed significant differences in the univariate analysis in the present study, but it failed to be a significant predictor.

In the present study, spontaneous resolution of stenosis was observed. Upon long-term follow-ups, 54.5% (12/22) of the cases showed improvement or complete resolution, while 36.4% of the cases remained stable. Lubicz et al. (22) also reported that 60% of the cases had improved or completely resolved stenosis and 28% of the cases were stable after Silk stenting during long-term follow-ups. In addition, most ISS after conventional stenting also improved during long-term follow-ups (30), suggesting that ISS after aneurysm stenting may be a dynamic benign course. Although most ISS have a benign prognosis, physicians should focus on aggravation cases, especially in cases with more than 50% stenosis. Two cases presented with symptomatic stenosis of acute cerebral thrombosis in the present study, with stenosis reaching 80% in one case and progression ranging from 65 to 95% in the other, suggesting that special attention and further follow-ups are needed for severe ISS.

This single-center retrospective study may have increased the risk of selection bias. Some patients underwent angiographic follow-ups in local hospitals, leading to some follow-up losses. Although the number of cohorts in this study was relatively large, further exploration of large-scale cohorts with long-term follow-up is needed. Despite these limitations, our research may provide more insights into planning proper treatment strategies when doctors encounter similar situations.

## Conclusions

In this retrospective study, the incidence of ISS was assessed, and the predictors of the severity of stenosis were determined through ordinal logistic regression. The results showed that ISS was a common angiographic finding after PED implantation and was presented as a largely benign course through long-term follow-up. Two cases presented with symptomatic stenosis, suggesting that special attention and further follow-up are needed for severe ISS. Patients with younger ages and longer procedure durations were at a greater risk of developing ISS.

## Data availability statement

The raw data supporting the conclusions of this article will be made available by the authors, without undue reservation.

## Ethics statement

Ethical review and approval was not required for the study on human participants in accordance with the local legislation and institutional requirements. Written informed consent from the patients/participants or patients/participants' legal guardian/next of kin was not required to participate in this study in accordance with the national legislation and the institutional requirements.

## Author contributions

YS, YJ, ZL, and YL contributed to the conception and design of the study. XC, DD, and YT organized the database. WY and JL performed the statistical analysis and wrote the draft of the manuscript. YS, ZL, and YJ performed the revision of the current literature. All authors contributed to the article and approved the submitted version.
